# Characterisation of FLT3 alterations in childhood acute lymphoblastic leukaemia

**DOI:** 10.1038/s41416-023-02511-8

**Published:** 2023-12-04

**Authors:** Angela Gutierrez-Camino, Chantal Richer, Manon Ouimet, Claire Fuchs, Sylvie Langlois, Fida Khater, Maxime Caron, Patrick Beaulieu, Pascal St-Onge, Alain R. Bataille, Daniel Sinnett

**Affiliations:** 1 Division of Hematology-Oncology, CHU Sainte-Justine Research Center, Montreal, Quebec Canada; 2 Department of Pediatrics, Faculty of Medicine, University of Montreal, Montreal, Quebec Canada

**Keywords:** Acute lymphocytic leukaemia, Cancer genetics

## Abstract

**Background:**

Alterations of *FLT3* are among the most common driver events in acute leukaemia with important clinical implications, since it allows patient classification into prognostic groups and the possibility of personalising therapy thanks to the availability of FLT3 inhibitors. Most of the knowledge on *FLT3* implications comes from the study of acute myeloid leukaemia and so far, few studies have been performed in other leukaemias.

**Methods:**

A comprehensive genomic (DNA-seq in 267 patients) and transcriptomic (RNA-seq in 160 patients) analysis of *FLT3* in 342 childhood acute lymphoblastic leukaemia (ALL) patients was performed. Mutations were functionally characterised by in vitro experiments.

**Results:**

Point mutations (PM) and internal tandem duplications (ITD) were detected in 4.3% and 2.7% of the patients, respectively. A new activating mutation of the TKD, G846D, conferred oncogenic properties and sorafenib resistance. Moreover, a novel alteration involving the circularisation of read-through transcripts (rt-circRNAs) was observed in 10% of the cases. Patients presenting *FLT3* alterations exhibited higher levels of the receptor. In addition, patients with *ZNF384*- and *MLL/KMT2A*-rearranged ALL, as well as hyperdiploid subtype, overexpressed FLT3.

**Discussion:**

Our results suggest that specific ALL subgroups may also benefit from a deeper understanding of the biology of *FLT3* alterations and their clinical implications.

## Introduction

The FMS-like tyrosine kinase 3 (*FLT3*) gene encodes a tyrosine kinase receptor primarily expressed in the bone marrow, in particular on CD34^+^ hematopoietic stem and early progenitor cells [1], which enables proliferation and differentiation [2]. Binding of the FLT3 ligand triggers its dimerisation and autophosphorylation, resulting in downstream activation of the PI3K/AKT, RAS/MAPK and STAT5-signalling pathways that lead to increased proliferation and reduced apoptosis [1, 3]. The high prevalence of *FLT3* -activating mutations in haematological malignancies, especially in acute myeloid leukaemia (AML), highlights the importance of this gene in leukemogenesis [2]. Most alterations occur in the juxtamembrane (JXM) and tyrosine kinase (TKD) domains [4]. Internal tandem duplications (ITDs) of the JXM domain disrupt the autoinhibitory function of the receptor, which leads to ligand-independent phosphorylation and activation. Point mutations (PM) in the TKD result in constitutive activation of the receptor [4, 5]. Rearrangements of *FLT3*, although rare, are reported in myeloid/lymphoid neoplasms with specific clinical characteristics [6–8]. Indeed, in vitro studies have shown that these rearrangements, especially *ETV6::FLT3*, causes ligand-independent dimerisation and downstream pathways activation [8]. The characterisation of these well-known aberrations at diagnosis has important clinical implications since it allows patients classification in prognostic groups, and offers the possibility of a personalised therapy thanks to the availability of FLT3 inhibitors [5]. However, most of the knowledge on *FLT3* implications in haematological malignancies comes from the study of AML. So far fewer studies have been performed in other leukaemias such as childhood acute lymphoblastic leukaemia (ALL).

Childhood ALL is the most frequent paediatric cancer, with cure rates now exceeding 80% due to concerted international efforts in treatment optimisation [9]. Despite remarkable improvements in survival, non-responding or relapsing patients still represent one of the most frequent causes of death by disease in children [9]. Therefore, the identification of actionable *FLT3* alterations could provide novel therapeutic approaches for high-risk patients. *FLT3* mutations have been identified in 3–6% of childhood ALL cases, with PM in the TKD being the most frequent alteration [5]. Some ALL subtypes (i.e., high hyperdiploid (HHD), *BCR::ABL1*-like (Ph-like)), exhibit a higher *FLT3* frequency of mutations [5, 10, 11] with several studies suggesting an association with prognosis [12, 13]. *FLT3* rearrangements have not been reported, but overexpression of the receptor is well described in some leukaemic subtypes regardless of mutational status, such as HHD [14] and *MLL/KMT2A*-rearranged ALL [15]. Interestingly, a recent study by Yang et al. [3] described a novel mechanism of FLT3 upregulation based on recurrent somatic microdeletions upstream *FLT3* and chromatin remodelling in HHD ALL, highlighting the importance of this gene as a driver in B-ALL. High levels of FLT3 have been associated with phosphorylation and ligand-independent activation in leukaemia [16], which makes patients with FLT3 overexpression susceptible for targeted therapy. All this work points to an important role of *FLT3* in childhood ALL pathogenesis, but further studies are still needed to draw conclusions with clinical impact. The aim of this study was to provide a comprehensive overview of *FLT3* alterations in childhood ALL as well as describe their functional involvement through the characterisation of a cohort of patients.

## Materials and methods

### Study population

The study included 342 children and adolescents from the well-characterised Quebec childhood ALL cohort (QcALL) [17] and DFCI-ALL Consortium Protocol 16-001 cohort [18]. Clinical and demographic data of patients, including age, sex, initial white blood cell (WBC) counts, and central nervous system (CNS) involvement, were collected at the time of enrolment (Table 1). In T-ALL patients, Early T-cell precursor (ETP) status was retrospectively determined by assessment of diagnostic flow cytometry data and was defined as the absence of CD1a and CD8, weak CD5 expression, and coexpression of myeloid and/or stem cell markers [19]. In addition, Minimal Residual Disease (MRD) at day 32 of induction 1A [18], 5-year event-free survival (EFS) and overall survival (OS) were collected from patients with enough follow-up. Six control samples (3 pre-B CD10 + /CD19+ and 3 pre-T CD3 + ) from six different donors sorted from the corresponding cell compartments of human cord blood were also included. Tumour material was obtained from bone marrow or peripheral blood collected at diagnosis. Genomic DNA and total RNA were extracted from patient’s tumour samples using mini or micro AllPrep DNA/RNA kits from Qiagen. The institutional review board approved the research protocol and written informed consent was obtained from all participants and their parents or legal guardians. The study was performed in accordance with the Declaration of Helsinki.

**Table 1:** Study population.

	Patients with DNA-seq dataset	Patients with RNA-seq dataset	Association with FLT3 alterations *P* value^ǂ^
Number of patients	267	160	–
Mean age ± SD, y	7.18 (4.6)	7.6 (4.6)	–
Patients < 1 y	5	1	n.s
Patients ≥ 1 < 10 y	183	106	n.s
Patients ≥ 10 y	79	53	n.s
Sex			
Male	148	90	n.s
Female	119	70	
Immunophenotype			
B	232	133	*
T	35	27	
Subtype classification in B-ALL			
ETV6::RUNX1/like	52	42	***
Hyperdiploid	77	34	***
TCF3::PBX1	5	4	n.s
DUX4-R	5	9	n.s
MLL/KMT2A-R	7	7	n.s
Ph + /Ph-like	11	15	n.s
PAX5 ALT	6	12	n.s
ZNF384-R	0	4	*
Hypodiploid	5	0	n.s
iAMP21	1	1	n.s
B-others/unknown	43	5	n.s
Subtype classification in T-ALL			
ETP-ALL^#^	2	4	n.s
WBC high (≥ 50 × 10^9^/L)	72	48	n.s
CNS > 1^#^	68	38	n.s
MRD high	17	38	n.s
Relapse^#^	38	16	n.s
Deceased	16	4	n.s

*SD* standard deviation, *WBC* white blood cells, *CNS* central nervous system involvement (CNS1 = CNS negative), *MRD* minimal residual disease, *n.s* non-significant.

^ǂ^Statistical analysis were performed with patients with RNA-seq datasets; ^#^ for some patients, clinical data is missing; comparisons assessed by Chi-square or Fisher exact test; *P value < 0.05; ***P value < 0.0005.

### Sequencing and data analysis

Whole exomes from 267 patients were sequenced as previously described [20, 21] and detailed in Supplementary information. RNA libraries from 160 patients were prepared from tumoral material using the Ribo-Zero Gold kit (Illumina) and the TruSeq Stranded Total RNA Library Prep Kit (Illumina) according to the manufacturer’s protocol. The resulting libraries were sequenced at ~150 million reads per samples (paired-end 2 × 75/2 × 100 bp) on HiSeq 2500/4000/NovaSeq 6000 sequencer. Sequencing was done at the Integrated Centre for Paediatric Clinical Genomics of the Centre Hospitalier Universitaire Sainte-Justine.

Bioinformatic analysis for whole exome sequencing (WES) data and whole transcriptome sequencing (RNA-seq) to call somatic single nucleotide variants and fusion genes was performed as described elsewhere [18, 20, 21] and detailed in Supplementary information. The mutational profile of *FLT3* was retrieved from the exome data and visualised using the Protein Paint tool [22]. We applied a leukaemia subtype classification tool to predict ALL subtypes using transcriptional signatures [18]. CICERO [23], a local assembly-based algorithm for RNA-seq data, was used to detect ITD in *FLT3*, selecting only those occurring in exon 14. CircExplorer [24] was used to identify circRNAs involving *FLT3*.

### Validation of genetic alterations

Y589D and G846D mutations were validated by Sanger sequencing. Validation of ITDs was performed by PCR as previously described [25]. Validation of read-through circRNAs (rt-circRNAs) was performed by RT-PCR with and without prior RNAse R treatment. Duplications were characterised by qPCR from genomic material, as previously described [26]. Primers, including divergent primers for rt-circRNAs, are listed in Supplementary Table 1.

### DNA constructs and vectors

The human FLT3-wild-type (WT) construct used as control was kindly provided by Karsten Spiekermann (University Hospital of Munich, Germany). The D835Y, Y589D and G846D mutations were introduced using the QuikChange II XL Site-Directed Mutagenesis Kit (Stratagene, La Jolla, CA). The construct sequences were confirmed by sequencing and transferred into pLenti-CMV-Puro-DEST vector using the Gateway system (ThermoFisher, Waltham, MA, USA).

### Cell lines, reagents and antibodies

Low-passage murine Ba/F3 cells, kindly provided by Connie J. Eaves (Terry Fox Laboratory, British Columbia Cancer Agency and University of British Columbia, Vancouver, BC, Canada), were cultured in RPMI-1640 (Wisent Bio Products, QC, Canada) supplemented with 10% FBS (Wisent Bio Products), 100 units/mL penicillin and streptomycin (Wisent Bio Products, QC, Canada) and 5 ng/ml of recombinant murine IL3 (Peprotech, NJ, USA) at 37 °C in a humidified atmosphere at 5% CO_2_. Cells infected with lentiviral plasmids were selected by adding puromycin (1 μg/mL) (Wisent Bio Products, QC, Canada) to the cell culture medium. Stable infection of Ba/F3 cells was performed as described previously [27]. Expression of *FLT3* was confirmed by RT-qPCR and Western blot as described elsewhere [28]. The following antibodies were used: anti-FLT3 (Cell Signaling, #3462, Danvers, MA, USA), anti-phospho ERK1/2 (Cell Signaling, #4370, Danvers, MA, USA), anti-ERK1/2 (Cell Signaling, #4695, Danvers, MA, USA), anti-phospho STAT5 (Cell Signaling, #9359, Danvers, MA, USA) and anti-STAT5a/b (Abcam, EPR16671-40, Cambridge, UK) and anti-GAPDH (sc-32233) from Santa Cruz Biotechnology (Santa Cruz, CA, USA).

### Ba/F3 transformation, cell growth and drug treatment assays

IL3-dependent Ba/F3 cells stably expressing the constructs were seeded at a concentration of 5 × 10^4^/mL in the presence or absence of IL3 as described elsewhere [29] in a 96-well plate. Cell proliferation was then measured by the CellTiter Glo assay (Promega, WI, USA) after 72 h. For drug treatment assays, Ba/F3 infected cells were treated for 48 h with various concentrations of either Sorafenib (ChemCruz Biochemicals, sc-220125; Lot #F0215, Santa Cruz, CA, USA) or Midostaurin (ChemCruz Biochemicals, sc-200691; Lot #K0718, Santa Cruz, CA, USA) or DMSO as control and cell viability was measured by CellTiter Glo.

### Statistical analysis

The association between *FLT3* alterations and patients´ characteristics was examined with Chi-square and Fisher exact tests. Survival curves were constructed by the Kaplan–Meier method. Since RNA-seq datasets allow the identification of PM, ITD, rt-circRNAs and expression, statistical analyses were performed in patients with RNA-seq data to avoid underestimation of *FLT3* alterations. FLT3 expression levels between subtypes and other clinical characteristics were compared using parametric tests after logarithmic transformation and normality testing using Shapiro–Wilk test. A *P* value ≤ 0.05 was considered statistically significant for all comparisons. Statistical analyses were done using GraphPad Prism 5.0 (GraphPad Software, CA, USA).

## Results

### Mutational landscape of FLT3

**Fig. 1:**
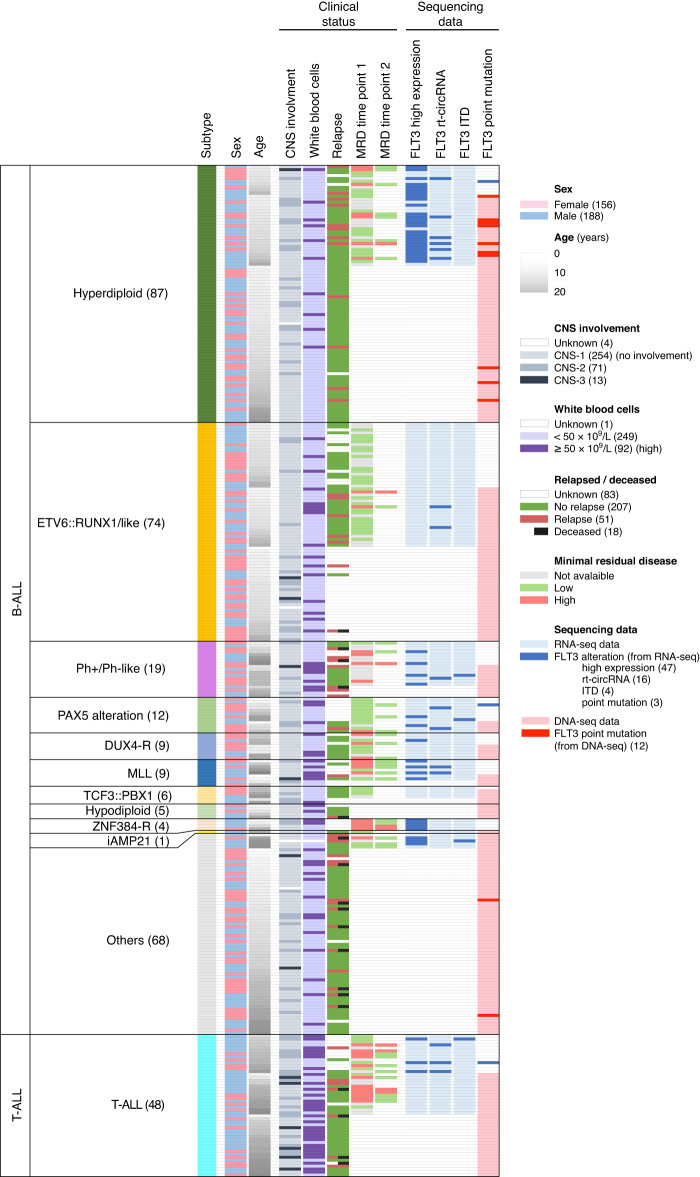
Overview of FLT3 study. A total of 342 patients with DNA-seq (*n* = 267) and RNA-seq (*n* = 160) data were included in the study. Sixteen PM in 15 patients were identified using both DNA-seq and RNA-seq data. An ITD in 4 patients and 8 rt-circRNAs in 16 patients were identified using RNA-seq data.

**Fig. 2:**
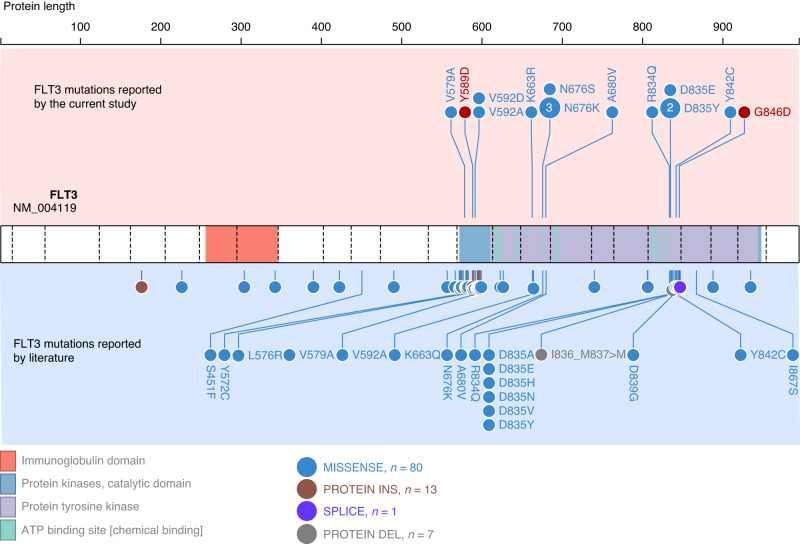
Diagram showing mutations reported in FLT3 in childhood ALL. In the upper part of the figure, FLT3 mutations reported in the current study. In red, mutations selected for functional validation. In the lower part, FLT3 mutations reported in literature. Fully displayed mutations (text) have been functionally characterised. Detail information about all previously reported mutations is available in Supplementary Tables 3 and 4.

The WES or RNA-seq data of 342 ALL patients were analysed to identify putative pathogenic somatic mutations in FLT3 (Fig. 1). We detected 16 PM (5 in DNA-seq only, 3 in RNA-seq only and 8 in both datasets) in 15 patients, with TKD mutations as the predominant alterations (Fig. 2 and Supplementary Table 2). Well-known activating mutation N676K in TKD1 was observed in three patients. Moreover, at positions D835 and V592, we observed two different amino acid substitutions (D835Y/D835E and V592A/V592D). From the 160 patients with available RNA-seq data, four presented an ITD in exon 14. ITD results were validated by conventional PCR for the two patients with sufficient available material. A higher number of mutations were associated with the Hyperdiploid subtype (11/15 patients) (Fig. 1). *FLT3* mutations in childhood ALL populations tend to be associated with relapse, and poor prognosis; thus, we analysed high-risk parameters in our cohort but found no association between *FLT3* mutations and age, initial WBC counts, MRD, CNS involvement or relapse (Table 1).

### Functional validation of recurrent mutations of FLT3

To select recurrent mutations for further functional studies, we performed an in-deep review of literature analysing *FLT3* mutations in the context of childhood ALL, and assessing the functional involvement of common alterations (Fig. 2). An exhaustive list of *FLT3* mutations described in childhood ALL is provided in Supplementary Table 3. In addition, mutations with functional information are listed with references in Supplementary Table 4. Based on our results and literature, we selected two recurring PM lacking functional characterisation: Y589D and G846D, two PM at the JXM and TKD, respectively. Both mutations were detected in our WES data confirmed in transcriptome data and validated by Sanger sequencing.

**Fig. 3:**
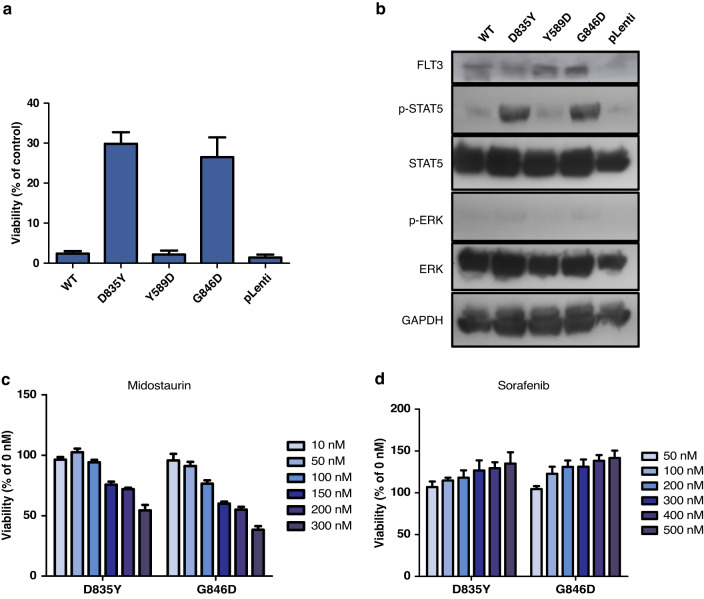
In vitro validation of FLT3 mutations. **a** Ba/F3 cells stably transduced with FLT3 WT, D835Y, Y589D, G846D and the empty vector were seeded at a density of 1 × 10^5^ in the presence or absence of IL3. Viable cells were assessed with CellTiter Glo assay after 72 h. Cell growth in the presence of IL3 was set to 100% for each cell line. Data represent values ± SD of triplicates. **b** Ba/F3 cells expressing the indicated constructs were starved overnight in media without IL3. STAT5 and ERK activation was analysed by Western blot from crude cell extract using 100 µg of proteins and phospho-specific antibodies, membranes were then stripped and reprobed with antibodies against total STAT5, ERK and GAPDH. For the visualisation of FLT3 receptor, 250 µg of proteins were used. Ba/F3 cells expressing FLT3 D835Y and G846D were incubated with increasing concentrations of **c** midostaurin and **d** sorafenib in the absence of IL3. Cell viability was determined after 48 h. Data are presented as percentage of untreated cells. Data represent mean values ± SD of triplicates.

The transforming potential of Y589D and G846D mutants was assessed using Ba/F3 cell line stably expressing several FLT3 constructs, including D835Y-FLT3 as a positive control and WT-FLT3 as negative control. RT-qPCR and western blot were used to confirm the stable expression of *FLT3* receptor for all constructs. G846D-FLT3 expression in cells led to IL3-independent growth (26%), while Y589D mutant failed to transform Ba/F3 cell line (Fig. 3a). The activation of downstream pathways, such as MAPK or STAT5 pathways, are critical for the transforming potential of *FLT3* mutants. Thus, we examined the phosphorylation status of two key signalling molecules downstream of FLT3: ERK and STAT5. Protein lysates from Ba/F3 cells expressing *FLT3* constructs were immunoblotted with specific antibodies against the phosphorylated and the unphosphorylated form of the proteins. Both G846D- and D835Y-mutants induced strong STAT5 phosphorylation, while Y589D mutant, WT-FLT3 and pLenti showed no activation. We observed no significant phosphorylation of the MAPK signalling cascade for any of the FLT3 constructs (Fig. 3b). Because of *FLT3* potential as a therapeutic target in childhood ALL, we tested the sensitivity of FLT3 mutants to Midostaurin, a type I *FLT3* inhibitor that binds to the active form of the receptor, and Sorafenib, a type II inhibitor which interacts with the inactive conformation [30]. FLT3-transformed Ba/F3 cells were grown in the presence of increasing concentrations of both inhibitors and cell viability was assessed after 48 h. Both G846D- and D835Y-mutants exhibited dose-dependent sensitivity to Midostaurin inhibition (Fig. 3c), with G846D-FLT3 being slightly more affected. Importantly, both mutants were resistant to Sorafenib treatment (Fig. 3d).

### Characterisation of fusion transcripts

**Fig. 4:**
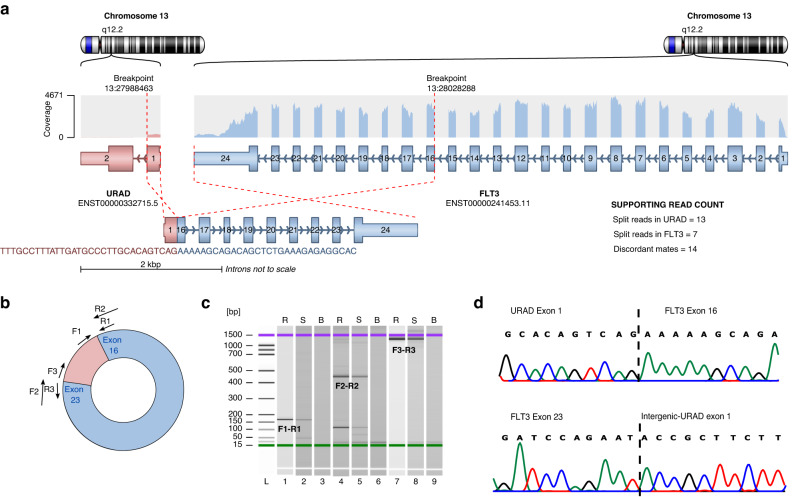
Characterisation of *rt-circURAD-FLT3*. **a** Fusion transcript *URAD::FLT3* containing *URAD*-exon 1 and exons 16–24 of *FLT3* as detected by FusionCatcher and Arriba from RNA-seq data. **b** Schematic showing the *rt-circURAD-FLT3* generated by the fusion transcript. Position of primers used for RT-PCR and Sanger sequencing are indicated by arrows. **c** Validation of the existence of *rt-circURAD-FLT3* in patient TC0133 by RT-PCR after RNAse R treatment, labelled R; cDNA generated using random priming, labelled S; cDNA generated using gene-specific priming, labelled B; blank. **d** Sanger sequencing confirms the fusion between 3´ exon 1 of *URAD* and 5´ exon 16 of *FLT3* and the backsplicing junction between 3´exon 23 of *FLT3* and 5´exon 1 of *URAD*.

Detection of fusion transcripts from RNA-seq data revealed a novel recurrent fusion event linking exon 1 of *URAD* and exon 16 of *FLT3* in frame (Fig. 4a) in two different patients (TC0133 and 871). No reciprocal product was detected. This result was validated by RT-PCR. *URAD* and *FLT3* are located ∼11 kb apart on chromosome 13, in the same orientation, indicating that the *URAD::FLT3* fusion transcripts may be the result of an intrachromosomal rearrangement at 13q12.2. Rearrangements involving exon 14 of *FLT3* have been described in myeloid/lymphoid malignancies for patients with high-risk prognosis [6, 7]. Both patients being classified as hyperdiploid B-ALL, were initially associated with good prognosis, but patient 871 presented CNS involvement and was thus reclassified as high risk and patient TC0133 relapsed, with the fusion transcript still present in relapse material. By cloning *URAD::FLT3* fusion gene in pLenti vectors and stably expressing this construct in Ba/F3 cells (data not shown), we failed to detect a fusion protein characteristic of *FLT3* rearrangements reported by other authors [7, 8]. This result prompted us to investigate alternative explanations that could lead to the observation of fusion transcripts by paired-end RNA sequencing [31].

The analysis for the presence of tandem duplications in the genomic material of patient TC0133 by qPCR was negative (data not shown). We next examined whether *URAD::FLT3* transcript resulted from the circularisation of read-through events, leading to the formation of a rt-circRNA, a novel class of circular transcripts [31]. In this scenario, the fusion event detected by FusionCatcher correspond to the backsplicing junction characteristic of circRNAs. RNA from patient TC0133 was digested with RNAse R to remove linear RNA and RT-PCR with primers diverging from the fusion point was performed (Fig. 4b). Visualisation of results on 2100 Bioanalyzer showed the amplification of a ∼1.3 Kb, RNAse R resistant, fragment, (Fig. 4c) matching the size of a transcript containing exon 1 of *URAD* and exons 16-23 of *FLT3*. Both the backspliced junction (exon 1 of *URAD*-exon 16 of *FLT3*) and read-through junction (exon 1 of *URAD*-exon 23 of *FLT3*) were identified by Sanger sequencing (Fig. 4d), demonstrating the existence of an rt-circRNA that include a small intergenic region between exon 1 of *URAD* and exon 23 of *FLT3* (Supplementary Table 5). To detect the presence of this new rt-circRNA in our cohort, we generated an artificial reference fusion gene, including exons 1–24 of *FLT3* and exon 1 of *URAD*. Among the 160 patients with RNA-seq data, we found 16 patients carrying several rt-circRNAs *URAD::FLT3*, 7 of them potentially containing the rt-circRNA characterised in patient TC0133, although with a low number of backspliced junctions. By correlation analysis we observed that patients with rt-circRNAs showed high levels of both *FLT3* and *URAD* genes (Supplementary Fig. 1). Importantly, 14 out 16 of the patients with rt-circRNAs presented characteristics associated with poor prognosis (Fig. 1 and Supplementary Table 6). None of the controls presented rt-circRNAs involving *FLT3* and *URAD* genes.

### FLT3 expression in childhood ALL

**Fig. 5:**
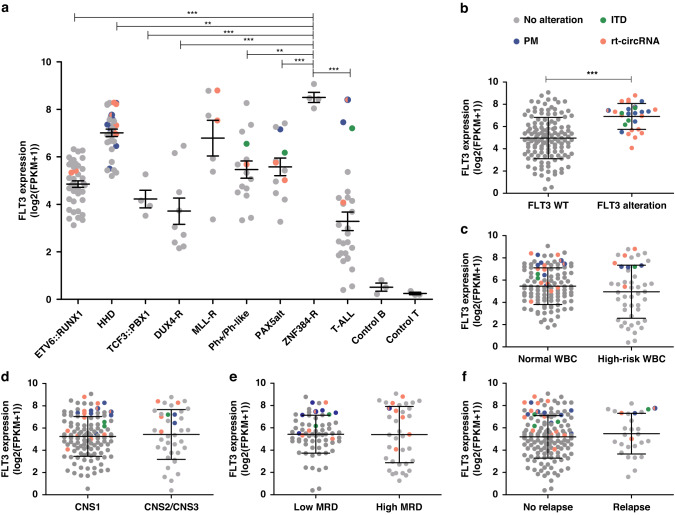
FLT3 expression. Box plots showing FLT3 gene expression levels measured by FPKM values extracted from transcriptome data and log-transformed by **a** ALL subtype, **b** FLT3 alterations, **c** white blood cell counts (considering high levels WBC ≥ 50×10^9^/L), **d** involvement of CNS, **e** MRD status at day 32 of induction, **f** relapse. The boxes extend from the 25th to 75th percentiles and the middle line represents median values. Comparisons assessed by ANOVA and *t* test, two sides. *P value < 0.01; **P value < 0.001; ***P value < 0.0001. Only significant *P* values (<0.05) are indicated.

FLT3 overexpression could lead to its activation as a receptor and be used as a prognostic marker in childhood ALL. We analysed FLT3 levels in the 160 patients with RNA-seq data. The mean expression of FLT3 in the whole cohort was 81.9 FPKM (range 0.3–536.4, median 39.3), with 47 patients showing a higher expression than the mean value (Fig. 1). We observed elevated FLT3 levels for all subtypes of B-ALL, as well as T-ALL, in comparison to controls. Interestingly, the highest mean overexpression was observed in *ZNF384*-rearranged ALL (373.9 FPKM), followed by *MLL/KMT2A*-rearranged (198.9 FPKM) and hyperdiploid ALL (152.3 FPKM) subtypes (Fig. 5a). In T-ALL, patients classified as ETP-ALL showed the highest levels of FLT3 compared to non-ETP cases (135.1 FPKM vs 14.4 FPKM). Considering the potential association between *FLT3* alterations and high FLT3 levels [5], we compared FLT3 expression in patients carrying mutations (PM/ITD) and/or rt-circRNAs and those without alterations. We found a statistically significant association between the expression level of the receptor and the alterations described (Fig. 5b). We observed no association of FLT3 expression with risk parameters, including WBC, CNS involvement, MRD status, or relapse (Fig. 5c–f). Moreover, survival curves indicated that *FLT3* aberrations, including mutations, rt-circRNAs and high levels of expression, were not associated with EFS and OS in our cohort (Supplementary Fig. 2).

## Discussion

In this study, we performed a comprehensive genomic, transcriptomic, literature review and functional characterisation of *FLT3* to investigate its alterations and their pathological impact in childhood ALL. PM were detected in 4.3% (15/342) of the patients and ITD in 2.5% (4/160) of them. Functional studies revealed a new activating mutation of the TKD, G846D, conferring oncogenic properties and sorafenib resistance. No *FLT3* translocations were detected, but we identified a novel alteration involving the circularisation of read-through transcripts observed in 10% (16/160) of the cases. Patients with *FLT3* mutations (PM/ITD) and/or rt-circURAD-FLT3 exhibited higher expression levels of the receptor compared to patients without alterations. In addition, 29.3% (47/160) of the patients showed high levels of FLT3, and patients with ZNF384-rearranged ALL subtype showed the highest overexpression of FLT3 compared to other subtypes and controls. Altogether, we observed FLT3 alterations in 35% (56/160) of our cohort.

*FLT3* mutations were detected in 19 patients, ITD or PM, with PM in the TKD being predominant. In agreement with previous data [5, 10], patients with Hyperdiploid subtype were overrepresented among the patients carrying *FLT3* mutations. Multiple studies observed high number of *FLT3* mutations in high-risk subtypes, such as Ph-like ALL [32, 33] or patients who relapse [32], as well as an association with age, including infant patients with *MLL/KMT2A* rearrangement and adults with early T-cell precursor ALL (ETP-ALL) [34, 35]. We and others found no association with risk markers [36, 37]. Among the PM reported in our cohort, we identified 4 well-known activating mutations in the kinase domain hotspot (D835-Y842), at residues D835Y/E, R834Q and Y842C. These mutations have been demonstrated to lead to constitutive phosphorylation of FLT3 protein and cell transformation, and are sensitive to FLT3 kinase inhibitors [38, 39]. We also detected a N676K mutation in 3 patients, recently shown to confer IL3-independent growth of the Ba/F3 cell line [40]. In addition, missense mutations were identified either close or within the JXM domain, including Y579A, V592A, V592D and K663R. Alterations at these positions were demonstrated to be activating mutations possibly reducing the stability of the inhibitory conformation of the JXM domain, making it more accessible for autophosphorylation [27, 38, 41]. Moreover, 2 unstudied mutations were also identified, Y589D and G846D, which were selected for further in vitro studies due to their recurrence in childhood ALL (Supplementary Table 3) and their location at key positions along FLT3 receptor.

Tyrosine residue Y589 is a phosphorylation site at the JXM domain critical for the ligand-dependent activation of native FLT3 and the transforming potential of oncogenic FLT3 mutants [29, 42]. Aspartic acid (D) is phosphomimetic of phospho-serine, which could favour the phosphorylated form, and thus activation. However, in the current study we observed no transformation capacity of Ba/F3 cells nor activation of downstream pathways, indicating that despite its high recurrence in childhood ALL, Y589D lacks oncogenic potential. A Y589E mutation, phosphomimetic of tyrosine, could have more impact because of the side chain conservation. G846 is located at the activation loop of TKD, where most validated mutations of *FLT3* are located [38, 39]. Indeed, our results showed that G846D induced IL3-independent growth and phosphorylation of STAT5, which indicates oncogenic potential. *FLT3* mutations may confer sensitivity to tyrosine kinase inhibitors, yet there appears to be substantial variation between mutations [28, 43] as well as between drugs [44, 45]. Type I inhibitors bind to the ATP-binding site when the receptor is active, while type II interact with a hydrophobic region immediately adjacent to the ATP-binding site only accessible when the receptor is in the inactive conformation [30]. Our results showed that G846D mutant is sensitive to type I inhibitor Midostaurin in a concentration-dependent manner, but resistant to type II inhibitor Sorafenib, which suggests that G846D mutation may favour the active conformation of FLT3. Indeed, several activating mutations of the TKD, mainly at position D835, are to various degrees insensitive to type II inhibitors [44, 45]. Our results add a new mutation to the list of actionable *FLT3* alterations with therapeutic potential in childhood ALL. This is of greater importance considering a recent study by Chougule et al. [46], which showed that secondary mutations in *FLT3* (ITD and R845G) are responsible for glucocorticoid resistance in ALL and displayed sensitivity to second-generation FLT3 inhibitors both in vitro and in vivo, suggesting that therapies targeting FLT3 might be useful for the treatment of B-ALL-relapsed patients.

We also identified a new type of alteration involving *FLT3*, the formation of rt-circRNAs, a novel class of circular transcripts resulting from read-through transcription of two adjacent and similarly oriented genes [47]. Recently, Vo et al. [31] described 1359 rt-circRNAs expressed in more than 2000 cancer and normal samples, 817 of them with cancer-specific expression. Despite the inclusion of 21 ALL samples in their cohort, none of the rt-circRNAs identified included *FLT3* as an acceptor or donor gene. Our results showed that *FLT3* produce different isoforms of rt-circRNAs with the same partner gene, *URAD*. The 5ʹ end of multiple *FLT3* exons are fused to the downstream 3ʹ end of exon 1 of *URAD*. The upregulation of *URAD* may result from transcriptional detour of gene transcriptional boundaries [48]. When canonical transcription termination is slowed or inhibited, the production of circRNAs is increased because their biogenesis can occur by read-through transcription [49]. Rt-circRNAs were more common in hyperdiploid patients and, importantly, most of them presented high-risk markers. So far, no functional studies have been performed to evaluate biological roles of rt-circRNAs, and the read-through transcription process is still understudied, but it has been suggested that they might affect the expression of their corresponding sense genes, be translated or function as miRNAs or RBPs sponges [47].

The most elevated levels of FLT3 were observed in patients with *ZNF384*-rearranged ALL, a new subtype of childhood ALL recently recognised as an independent entity [9]. Mutations in the FLT3 receptor were previously reported in this ALL subtype [50, 51], but none of our patients presented any alteration in this gene (PM, ITD, rt-circRNA). Interestingly, Alexander et al. [52], also identified the highest overexpression of FLT3 in patients with mixed phenotype acute leukaemia (MPAL) and *ZNF384* alteration regardless of the mutational status, indicating that it is a hallmark of the rearrangement. Indeed, Zhao et al. [53] recently revealed an intergenic enhancer element at the *FLT3* locus that is exclusively activated in *ZNF384*-rearranged ALL, directly mediated by the fusion protein. High levels of FLT3 were also observed in *MLL/KMT2A*, Hyperdiploid and ETP-ALL patients, as reported in previous studies [14, 15, 54], although no differences with other risk parameters or relapse were found. There is little information about the prognostic significance of FLT3 expression in distinct subgroups of ALL, but multiple studies demonstrated an association when the analysis was restricted to *MLL/KMT2A*-rearranged subtype [12, 36]. In our study, all patients with *ZNF384*-rearranged ALL presented high levels of MRD at day 32 of induction, suggesting that further studies in this subtype could refine the prognostic impact of FLT3 expression. Upregulation of FLT3 in ALL could represent an opportunity to improve disease outcome using FLT3 inhibitors. Indeed, studies analysing drug responsiveness in *ZNF384-* and *MLL/KMT2A*-childhood ALL [5, 55], suggested that FLT3 targeting therapy could be considered in these forms of leukaemia, especially in *ZNF384-*ALL. Other high-risk subtypes of ALL with high FLT3 levels could also be eligible for FLT3 therapies, like the recently described ETP-ALL [56]. For this purpose, it is critical to define a well-established cut-off value to accurately categorise patients into a group with FLT3 overexpression [5], and establish a proper method to evaluate FLT3 levels in a clinical setting. The presence of rt-circURAD-FLT3 transcripts in high-risk patients and their association with high levels of FLT3 expression could make them valuable markers for FLT3 overexpression.

In conclusion, although fewer studies looking at *FLT3* have been performed on ALL than on AML, our results in combination with recent literature suggest that specific ALL subtypes and subgroups may also benefit from a deeper understanding of the biology of *FLT3* alterations and their clinical implications. Most of the knowledge derived from the study of *FLT3* in AML, is transposable to childhood ALL, but it remains important to characterise the alterations specific to ALL and determine their clinical relevance. A combination of genetic and functional approaches is crucial to identify mutations susceptible to FLT3 inhibitors and evaluate their sensitivity to select the best treatment. The determination of high-risk patients who could benefit the most from new therapies in an already successful treatment protocol is also pending. For patients presenting glucocorticoid resistance, relapse patients, and patients classified with poor prognosis subtypes, the use of specific FLT3 inhibitors could improve disease outcomes.

### Supplementary information

Supplementary Material

Supplementary Table 2

## Data Availability

The data related to this article are available upon request from the corresponding author.
