# Supplementary material for: Characterisation of FLT3 alterations in childhood acute lymphoblastic leukaemia
Source: Br J Cancer. 2023 Dec 4;130(2):317–26. doi: 10.1038/s41416-023-02511-8 (PMC10803556; doi:10.1038/s41416-023-02511-8)
Supplement: Supplementary file 1 — Supplementary Material [file 41416_2023_2511_MOESM1_ESM.pdf]

Characterization of FLT3 alterations in childhood acute lymphoblastic leukemia

Running title: FLT3 in childhood ALL

Angela Gutierrez-Camino[1], Chantal Richer[1], Manon Ouimet[1], Claire Fuchs[1], Sylvie Langlois[1], Fida Khater[1], Maxime Caron[1], Patrick Beaulieu[1], Pascal St-Onge[1], Alain R Bataille[1], Daniel Sinnett[1,2,*]

[1]Division of Hematology-Oncology, CHU Sainte-Justine Research Center, Montreal, Canada, [2]Department of Pediatrics, Faculty of Medicine, University of Montreal, Montreal, Canada.

[*]Corresponding author:

Daniel Sinnett, PhD, Centre Hospitalier Universitaire Sainte-Justine, 3175 Chemin de la Côte-Sainte-Catherine, Montréal, Québec, Canada H3T 1C5 (daniel.sinnett@umontreal.ca) (ORCID: 0000-0003-3625-6676)

Supplementary Materials and Methods

Whole exome sequencing and RNA-seq

Whole exomes from 267 patients were captured using SureSelect XT Clinical Research Exome (n=185, Agilent) and Nextera Rapid Capture Exome Enrichment (n=80, Illumina) kits according to the manufacturers' instructions. Single-end (average 32X coverage, 75 base pairs (bp)) and paired-end sequencing (average 200X, 2 × 75 bp) of tumor materials was performed on the sequencing system SOLID (Life Technologies), and HiSeq 2500/4000/NovaSeq 6000 (Illumina), respectively. RNA libraries from 160 patients were prepared from tumoral material using the Ribo-Zero Gold kit (Illumina) and the TruSeq Stranded Total RNA Library Prep Kit (Illumina) according to the manufacturer's protocol. The resulting libraries were sequenced at approximately 150 million reads per samples (paired-end 2 × 75 bp or 2 × 100 bp) on HiSeq 2500/4000/NovaSeq 6000 sequencer. Sequencing was done at the Integrated Centre for Pediatric Clinical Genomics of the Centre Hospitalier Universitaire Sainte-Justine.

Data analysis of WES

Reads obtained from SOLiD and HiSeq 2500/4000/NovaSeq 6000 systems were aligned to the hg19 reference genome using LifeScope Genomic Analysis Software and Bowtie2 (1) respectively. Picard (http://picard.sourcefourge.net) was used to remove duplicate mappings, calculate metrics, and manipulate SAM/BAM files. Genotype quality score recalibration was performed using the Genome Analysis ToolKit (GATK) (2). After filtering out low quality reads, pileup files were created using SAMtools (3). Somatic single nucleotide variants (SNVs) and small indels were called using Varscan2 (4) and MuTect.(5) In addition, the resulting somatic mutations were screened against 1000 Genomes 10, NHLBI ESP data (evs.gs.washington.edu/EVS/), and our in-house database of normal exomes to filter out variants with minor allele frequency >0.01. To enrich for putative pathogenic driver variants, the predicted functional impact of non-synonymous variants and small indels were assessed using Sift (6) and Polyphen2 (7). Any variants classified as benign or likely benign were excluded. Raw data of FLT3 variants is provided in Supplementary Table 2.

Data analysis of RNA-seq data

For whole transcriptome sequencing (RNA-seq), alignment to the hg19 (GRCh37) genome reference was performed using STAR (8). We applied a leukemia subtype classification tool to predict ALL subtypes using transcriptional signatures, as previously described (9). Haplotypecaller from the Genome Analysis Toolkit (GATK) was used for SNV calling. Filtered-based depth of coverage >30 and allele frequency of <1% in any of the subpopulations of the 1000 Genomes Project database were applied; mutations listed in COSMIC were identified. Novel mutations lying in hotspots and those predicted to be pathogenic by SIFT and Polyphen2 were also reported. Fusion genes, translocations and chimeric transcripts were identified using Arriba (10) and FusionCatcher (11). STAR Fusion(12) was used to rank putative reciprocal breakpoints from the STAR output. CICERO (13), a local assembly-based algorithm for RNA-seq data, was used to detect internal tandem duplications in *FLT3*, selecting only those occurring in exon 14. CircExplorer (14) was used to identify circRNAs involving *FLT3*. Fragments per kilobase per million mapped reads (FPKM) values for FLT3 were obtained using the cufflinks software on the Ensembl version 75 gene annotation (15).

Supplementary Figures

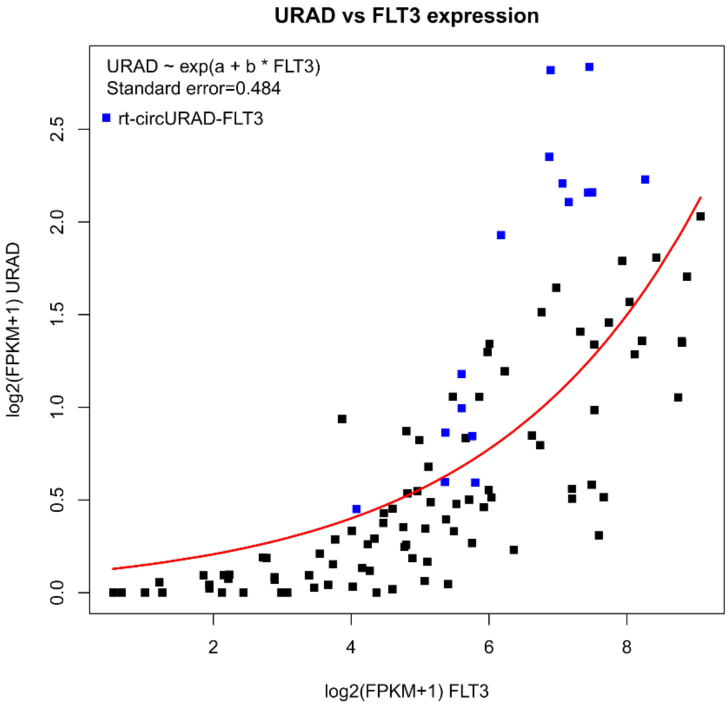

Supplementary Figure 1: Representation of FLT3 and URAD gene expression levels measure by FPKM values extracted from transcriptome data and log transformed. Highlighted in blue those patients carrying rt-circRNAs.

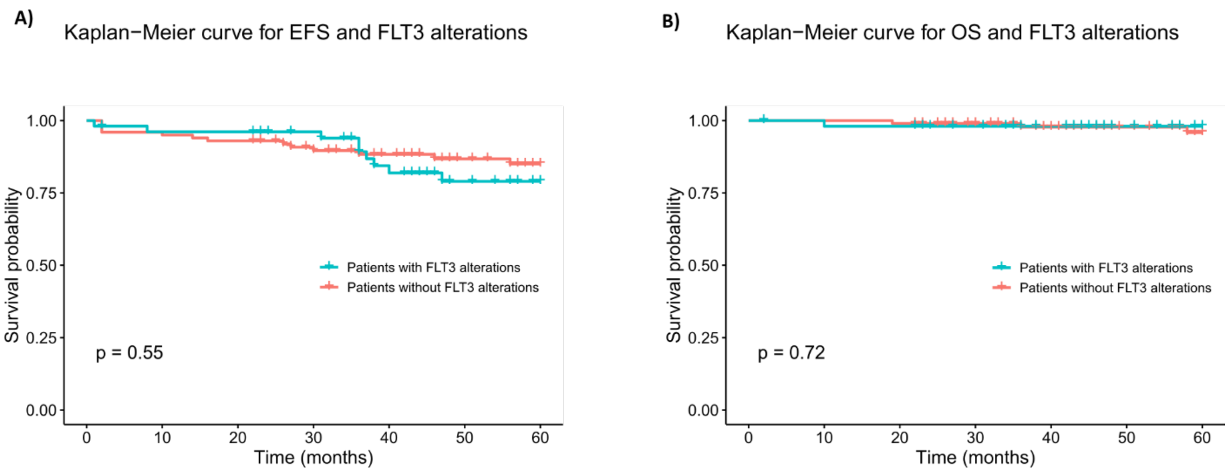

Supplementary Figure 2: Kaplan-Meier curves representing **A)** 5-years Event Free Survival (EFS) and **B)** Overall Survival (OS) in patients with and without FLT3 alterations. EFS was defined as time from the start date of study treatment to the time when primary refractory disease was confirmed (ie, the date when failure to achieve a response to induction therapy was determined), relapse, or death. Patients last known to be alive without an event were censored at the date of last contact. OS was defined from the date of registration to the study to the date of death from any cause, with patients last known to be alive censored at the date of last contact.

Supplementary tables

Supplementary Table 1: List of primers used in the current study

Gene	***Forward***	***Reverse***
ITD FLT3	GCAATTTAGGTATGAAAGCCAGC	CTTTCAGCATTTTGACGGCAACC
CNV FLT3	CAGCTCTGAAAGAGAGGCACT	ACAATATTCTCGTGGCTTCCCA
CNV URAD	TGTTTGGGAATGCCACTGAGA	CAAATCAGAGAATGGCCGCTG
Rt-circRNA (BSJ)	ATGTGTTTGGGAATGCCACT	TGCCTCTCTTTCAGAGCTGTC
Rt-circRNA (DSJ)	CGGCCATCCTTCCCTAATTTGA	TCTTGAGTTCTGACATGAGTGCC
Rt-circRNA (full length)	TTGCACAGTCAGAAAAAGCAGAC	GGGCATCAATAAAGGCAAAAAAGTG

Abbreviations: BSJ: Back splice junction, DSJ: Double splice junction

Supplementary Table 3: FLT3 mutations reported in childhood ALL by literature

Mutation	*Reference*
T227M	Zakaria Z Asian Pac J Cancer Prev. 2017(16)
T305I	Ding LW Cancer Res 2017(17)
T343I	Roberts N Engl J Med. 2014(18)
R391S	Stam RW Haematologica. 2007(19)
E421K	Ding LW Cancer Res 2017(17)
S451F	Malinowska-Ozdowy K Leukemia 2015(20)
V491L	Zhang Blood 2011(21); Liu Y Nat Genet. 2017(22); Ding LW Cancer Res 2017(17)
V557I	Stam RW Haematologica. 2007(19)
K567I	Stam RW Haematologica. 2007(19)
Y572C	Zhang Blood 2011(21), Malinowska-Ozdowy K Leukemia 2015(20)
E573G	Zhang Y Cancer Gene Ther. 2020(23)
Q575P	Zhang Y Cancer Gene Ther. 2020(23)
L576P	Holmfeldt L, Nat Genet 2013(24); Zhang H, BMC Cancer. 2020(25); Ding LW Cancer Res 2017(17); Zhang Nat Genet 2016(26)
L576Q	Zhang Y Cancer Gene Ther. 2020(23); Malinowska-Ozdowy K Leukemia 2015(20)
L576R	de Smith AJ Oncotarget 2016(27)
V579A	Lindqvist CM Oncotarget 2016(28); Zhang Y Cancer Gene Ther. 2020(23)
V579E	Lindqvist CM Oncotarget 2016(28)
V579G	Reshmi SC Blood 2017(29); Zhang Y Cancer Gene Ther. 2020(23)
V579Q	Stam RW Haematologica. 2007(19)
Q580P	Zhang Y Cancer Gene Ther. 2020(23)
T582P	Malinowska-Ozdowy K Leukemia 2015(20); Paulsson Nat Genet 2015(30)
G583S	Zhang Y Cancer Gene Ther. 2020(23)
Y589D	Paulsson Nat Genet 2015(30); Zhang H, BMC Cancer. 2020(25); Zhang Y Cancer Gene Ther. 2020(23); Ding LW Cancer Res 2017(17); Malinowska-Ozdowy K Leukemia 2015(20)
Y589H	Zhang H, BMC Cancer. 2020(25)
F590fs	Zhang H, BMC Cancer. 2020(25)
F590L	Zhang Y Cancer Gene Ther. 2020(23)
Y591D	Malinowska-Ozdowy K Leukemia 2015(20)
Y591H	Lindqvist CM Oncotarget 2016(28)
V592A	Zhang H, BMC Cancer. 2020(25); Zhang Y Cancer Gene Ther. 2020(23)
V592D	Paulsson Nat Genet 2015(30); Ding LW Cancer Res 2017(17)
V592E	Zhang H, BMC Cancer. 2020(25)
V592F	Zhang H, BMC Cancer. 2020(25)
F594I	Zhang Blood 2011(21)
F594V	Zhang Y Cancer Gene Ther. 2020(23); de Smith AJ Oncotarget 2016(27)
Y599C	Zhang Y Cancer Gene Ther. 2020(23); de Smith AJ Oncotarget 2016(27)
K623I	Malinowska-Ozdowy K Leukemia 2015(20); Zhang Y Cancer Gene Ther. 2020(23)
A627T	Zhang Y Cancer Gene Ther. 2020(23)
K663Q	Lindqvist CM Oncotarget 2016(28); Zhang Nat Genet 2016(26)
K663R	Chen C, Leuk Res 2015(31); Malinowska-Ozdowy K Leukemia 2015(20); de Smith AJ Oncotarget 2016(27); Ding LW Cancer Res 2017(17)
M664I	de Smith AJ Oncotarget 2016(27)
M664V	Malinowska-Ozdowy K Leukemia 2015(20)
N676K	de Smith AJ Oncotarget 2016(27); Zhang Blood 2011(21); Ding LW Cancer Res 2017(17); Andersson Nat Genet 2015(32)
A680V	Ishida H, Pediatr Blood Cancer. 2016; Ding LW Cancer Res 2017(17); de Smith AJ Oncotarget 2016(27); Roberts N Engl J Med. 2014(18); Malinowska-Ozdowy K Leukemia 2015(20)
S740L	Ding LW Cancer Res 2017(17)
S806L	Safavi Oncotarget 2016(33)

Mutation	**Reference**
R810G	Malinowska-Ozdowy K Leukemia 2015(20)
R834Q	de Smith AJ Oncotarget 2016(27)
D835	Zhang Blood 2011(21)
D835A	Zhang H, BMC Cancer. 2020(25)
D835E	Zhang Y Cancer Gene Ther. 2020(23)
D835H	Lindqvist CM Oncotarget 2016(28); Paulsson Nat Genet 2015(30)
D835N	Lindqvist CM Oncotarget 2016(28)
D835V	Malinowska-Ozdowy K Leukemia 2015(20); Roberts N Engl J Med. 2014(18); de Smith AJ Oncotarget 2016(27)
D835Y	Spinella JF, BMC Cancer 2015(34); Zhang Nature. 2012(35); Zhang Nat Genet 2016(26); Roberts N Engl J Med. 2014(18); Zhang Y Cancer Gene Ther. 2020
I836F	Ding LW Cancer Res 2017(17)
I836N	de Smith AJ Oncotarget 2016(27)
M837K	Zhang Y Cancer Gene Ther. 2020(23)
D839G	Zhang Y Cancer Gene Ther. 2020(23); Andersson Nat Genet 2015(32)
N841T	Malinowska-Ozdowy K Leukemia 2015(20); Zhang Y Cancer Gene Ther. 2020(23)
Y842C	de Smith AJ Oncotarget 2016(27); Ding LW Cancer Res 2017(17)
Y842N	Zhang Y Cancer Gene Ther. 2020(23)
Y842S	Zhang H, BMC Cancer. 2020(25)
V843D	Malinowska-Ozdowy K Leukemia 2015(20)
R845G	Zhang H, BMC Cancer. 2020(25)
G846A	Holmfeldt L, Nat Genet 2013(24)
I867S	Malinowska-Ozdowy K Leukemia 2015(20)
P888S	Ding LW Cancer Res 2017(17)
P934L	Andersson Nat Genet 2015(32)

Supplementary Table 4: FLT3 mutations with experimental evidence

Mutation	*Domain*	*Function*	*Disease*	*Reference*
T167A	Extracelular	Passenger	AML	Fröhling S, et al., Cancer Cell, 2007(36)
V194M	Extracelular	Passenger	AML	Fröhling S., et al., Cancer Cell, 2007(36)
Y364H	Extracelular	Passenger	AML	Fröhling S., et al., Cancer Cell, 2007(36)
S451F	Extracelular	Gain-of-Function	AML	Fröhling S., et al., Cancer Cell, 2007(36)
S471C	Extracelular	Gain-of-Function	ALL	Vicente et al., Haematologica, 2009(37)
Q569Vfs*2	Juxtamembrane	Loss-of-function	AML	Sandhöfer N., et al., Sci Rep, 2016(38)
Y572C	Juxtamembrane	Gain-of-Function	AML	Fröhling S., et al., Cancer Cell, 2007(36)
E573D	Juxtamembrane	Receptor phosphorylation	AML	Tarlock et al., Blood, 2015*
L576R	Juxtamembrane	Receptor phosphorylation	AML	Tarlock et al., Blood, 2015*
V579A	Juxtamembrane	Gain-of-Function	AML	Reindl C, et al., Blood 2006(39)
F590_Y591delinsGD	Juxtamembrane	Gain-of-Function	AML	Reindl C, et al., Blood 2006(39)
V592A	Juxtamembrane	Gain-of-Function	AML	Reindl C, et al., Blood 2006(39)
V592G	Juxtamembrane	Gain-of-Function	AML	Fröhling S., et al., Cancer Cell, 2007(36)
F594L	Juxtamembrane	Gain-of-Function	AML	Reindl C, et al., Blood 2006(39)
Y599C	Juxtamembrane	Receptor phosphorylation	AML	Tarlock et al., Blood, 2015*
D600H	Juxtamembrane	Gain-of-Function	AML	Andersson et al., Nat Gen 2015(32)
D600G	Juxtamembrane	Receptor phosphorylation	AML	Tarlock et al., Blood, 2015*
K663Q	TKD1	Gain-of-Function	AML	Schittenhelm et al., Leukemia, 2006(40)
A680V	TKD1	Receptor phosphorylation	AML	Tarlock et al., Blood, 2015*
N676K	TKD1	Gain-of-Function	AML	Opatz S, et al., Blood, 2013(41); Andersson et al., Nat Gen 2015(32)
M737I	TKD1	Passenger	AML	Fröhling S., et al., Cancer Cell, 2007(36)
G831E	TKD2	Passenger	AML	Fröhling S., et al., Cancer Cell, 2007(36)
R834Q	TKD2	Gain-of-Function	AML	Fröhling S., et al., Cancer Cell, 2007(36)
D835Y	TKD2	Gain-of-Function	AML	Yamamoto et al., Blood, 2001(42)
D835H	TKD2	Gain-of-Function	AML	Yamamoto et al., Blood, 2001(42)
D835V	TKD2	Gain-of-Function	AML	Yamamoto et al., Blood, 2001(42)
D835E	TKD2	Gain-of-Function	AML	Yamamoto et al., Blood, 2001(42)
D835N	TKD2	Gain-of-Function	AML	Yamamoto et al., Blood, 2001(42)
D835A	TKD2	Gain-of-Function	AML	Clark et al., Blood, 2004(43)
I836M+R	TKD2	Gain-of-Function	AML	Grundler R et al., Blood 2003(44)
I836L+D	TKD2	Gain-of-Function	AML	Yamamoto et al., Blood, 2001(42)
I836del	TKD2	Gain-of-Function	AML	Grundler R et al., Blood 2003(44); Armstrong et al., Cancer Cell, 2003(45)
D839G	TKD2	Gain-of-Function	AML	Janke et al., Plos One, 2014(46); Andersson et al., Nat Gen 2015(32)
840GS	TKD2	Gain-of-Function	AML	Spiekermann et al., Blood, 2002(47)
N841I	TKD2	Gain-of-Function	AML	Jiang et al., Blood, 2004(48)
Y842H	TKD2	Gain-of-Function	AML	Bagrintseva et al., Blood, 2004(49)
Y842C	TKD2	Gain-of-Function	AML	Kindler et al., Blood 2005(50)
I867S	TKD2	Gain-of-Function	AML	Janke et al., Plos One, 2014(46)
K868N	TKD2	Loss-of -function	AML	SERIZAWA et al., Anticancer Res, 2016
P934L	TKD2	No activating mutations	AML	Andersson et al., Nat Gen 2015(32)

Supplementary Table 5: Sequence of rt-circURAD-FLT3 identified in patient TC0133

rt-circURAD-FLT3	AAAAAGCAGACAGCTCTGAAAGAGAGGCACTCATGTCAGAACTCAA<br>GatgatgacccagctgggaagccacgagaatattgtgaacctgctgggggcgtgcacactgtcagGACCA<br>ATTTACTTGATTTTTGAATACTGTTGCTATGGTGATCTTCTCAACTAT<br>CTAAGAAGTAAAAGAGAAAAATTTCACAGGACTTGGACAGAGATTT<br>TCAAGGAACACAATTTCAGTTTTTACCCCACTTTCCAATCACATCCA<br>AATTCCAGCATGCCTGGTTCAAGAGAAGTTCAGATACACCCGGACTC<br>GGATCAAATCTCAGGGCTTCATGGGAATTCATTTCACTCTGAAGATG<br>AAATTGAATATGAAAACCAAAAAAGGCTGGAAGAAGAGGAGGACT<br>TGAATGTGCTTACATTTGAAGATCTTCTTTGCTTTGCATATCAAGTTG<br>CCAAAGGAATGGAATTTCTGGAATTTAAGTCGTGTGTTCACAGAGAC<br>CTGGCCGCCAGGAACGTGCTTGTCACCCACGGGAAAGTGGTGAAGA<br>TATGTGACTTTGGATTGGCTCGAGATATCATGAGTGATTCCAACTAT<br>GTTGTCAGGGGCAATGCCCGTCTGCCTGTAAAATGGATGGCCCCCGA<br>AAGCCTGTTTGAAGGCATCTACACCATTAAGAGTGATGTCTGGTCAT<br>ATGGAATATTACTGTGGGAAATCTTCTCACTTGGTGTGAATCCTTAC<br>CCTGGCATTCCGGTTGATGCTAACTTCTACAAACTGATTCAAAATGG<br>ATTTAAAATGGATCAGCCATTTTATGCTACAGAAGAAATAtacattataatg<br>caatcctgctgggcttttgactcaaggaaacggccatccttccctaatttgacttcgtttttaggatgtcagctggcag<br>atgcagaagaagcggtattctggatctgcttctcccaaaggcatttaaaacacgtgaaggtggtgttgcgtaaaca<br>gcaagctcttgtgttcagcagatataaccgagtgagccgagacagcgtcactcactcaccgagaggggagctgg<br>acgtcccgctgtctccagtggaatacaaggaATGGACATTGAGAAGGTCAACTCCATG<br>GACCTTGGAGAATTCGTGGATGTGTTTGGGAATGCCACTGAGAGATG<br>TCCTCTGATTGCAGCTGCTGTTTGGTCCCAGCGGCCATTCTCTGATTT<br>GGAAGATTTAGAGAAGCACTTTTTTGCCTTTATTGATGCCCTTGCAC<br>AGTCAG

Supplementary Table 6: Characteristics of patients with rt-circURAD-FLT3

Sample	*Exon of FLT3 involved*	*Poor prognosis characteristic*
SISJ0420	Exon 16 (2 BSJ reads)	WBC ≥50x10^9
16-114	Isoform 1: Exon 5 (2 BSJ reads)<br>Isoform 2: Exon 3 (1 BSJ reads)	Pre-T ALL (ETP-ALL), age≥10, CNS2
871	Isoform 2: Exon 16 (3 BSJ reads)	CNS2
995	Exon 16 (1 BSJ reads)	Ph+/Ph-like, age≥10, CNS2
TC0133	Exon 16 (26 BSJ reads)	Relapse
16-161	Exon 6 (2 BSJ reads)	Relapse
16-282	Exon 12 (5 BSJ reads)	CNS2
16-273	Exon 16 (2 BSJ reads)	WBC ≥50x10^9
16-170	Exon 16 (1 BSJ reads)	Relapse
SISJ0403	Exon 12 (1 BSJ reads)	MRD high at TP 1
SISJ0400	Exon 20 (1 BSJ reads)	-
16-039	Exon 20 (1 BSJ reads)	-
16-042	Exon 21 (1 BSJ reads)	Pre-T ALL
16-098	Isoform 1: Exon 6 (3 BSJ reads)<br>Isoform 2: Exon 16 (1 BSJ reads)	Pre-T ALL (ETP-ALL), age≥10
16-132	Exon 6 (1 BSJ reads)	MLL, age≥10, WBC ≥50x10^9
16-038	Isoform 1: Exon 2 (1 BSJ reads)<br>Isoform 2: Exon 16 (5 BSJ reads)	MLL, age≥10, WBC ≥50x10^9

Abbreviations: BSJ Back Splicing Junction; WBC White Blood Cells; CNS Central Nervous System involvement (CNS1=CNS negative); MRD minimal Residual Disease; TP time point

References

1. Langmead B, Salzberg SL. Fast gapped-read alignment with Bowtie 2. Nat Methods. 2012 Mar 4;9(4):357-9.
2. McKenna A, Hanna M, Banks E, Sivachenko A, Cibulskis K, Kernytsky A, et al. The Genome Analysis Toolkit: a MapReduce framework for analyzing next-generation DNA sequencing data. Genome Res. 2010 Sep;20(9):1297-303.
3. Li H, Handsaker B, Wysoker A, Fennell T, Ruan J, Homer N, et al. The Sequence Alignment/Map format and SAMtools. Bioinformatics. 2009 Aug 15;25(16):2078-9.
4. Koboldt DC, Chen K, Wylie T, Larson DE, McLellan MD, Mardis ER, et al. VarScan: variant detection in massively parallel sequencing of individual and pooled samples. Bioinformatics. 2009 Sep 1;25(17):2283-5.
5. Cibulskis K, Lawrence MS, Carter SL, Sivachenko A, Jaffe D, Sougnez C, et al. Sensitive detection of somatic point mutations in impure and heterogeneous cancer samples. Nat Biotechnol. 2013 Mar;31(3):213-9.
6. Ng PC, Henikoff S. SIFT: Predicting amino acid changes that affect protein function. Nucleic Acids Res. 2003 Jul 1;31(13):3812-4.
7. Adzhubei I, Jordan DM, Sunyaev SR. Predicting functional effect of human missense mutations using PolyPhen-2. Curr Protoc Hum Genet. 2013 Jan;Chapter 7:Unit7 20.
8. Dobin A, Davis CA, Schlesinger F, Drenkow J, Zaleski C, Jha S, et al. STAR: ultrafast universal RNA-seq aligner. Bioinformatics. 2013 Jan;29(1):15-21.
9. Tran TH, Langlois S, Meloche C, Caron M, St-Onge P, Rouette A, et al. Whole-transcriptome analysis in acute lymphoblastic leukemia: a report from the DFCI ALL Consortium Protocol 16-001. Blood Adv. 2021 Dec 21.
10. Uhrig S, Ellermann J, Walther T, Burkhardt P, Frohlich M, Hutter B, et al. Accurate and efficient detection of gene fusions from RNA sequencing data. Genome Res. 2021 Mar;31(3):448-60.
11. Nicorici D, Şatalan M, Edgren H, Kangaspeska S, Murumägi A, Kallioniemi O, et al. FusionCatcher – a tool for finding somatic fusion genes in paired-end RNA-sequencing data. BioRxiv 2014;011650:doi.org/10.1101/011650.
12. Haas BJ, Dobin A, Stransky N, Li B, Yang X, Tickle T, et al. STAR-Fusion: Fast and Accurate Fusion Transcript Detection from RNA-Seq. BioRxiv. 2017;120295:doi.org/10.1101/120295.

13. Tian L, Li Y, Edmonson MN, Zhou X, Newman S, McLeod C, et al. CICERO: a versatile method for detecting complex and diverse driver fusions using cancer RNA sequencing data. Genome Biol. 2020 May 28;21(1):126.
14. Ma XK, Wang MR, Liu CX, Dong R, Carmichael GG, Chen LL, et al. CIRCexplorer3: A CLEAR Pipeline for Direct Comparison of Circular and Linear RNA Expression. Genomics Proteomics Bioinformatics. 2019 10;17(5):511-21.
15. Trapnell C, Williams BA, Pertea G, Mortazavi A, Kwan G, van Baren MJ, et al. Transcript assembly and quantification by RNA-Seq reveals unannotated transcripts and isoform switching during cell differentiation. Nat Biotechnol. 2010 May;28(5):511-5.
16. Zakaria Z, Othman N, Ismail A, Kamaluddin NR, Esa E, Abdul Rahman EJ, et al. Whole-Exome Sequencing of ETV6/RUNX1 in Four Childhood Acute Lymphoblastic Leukaemia Cases. Asian Pac J Cancer Prev. 2017 Apr 1;18(4):1169-75.
17. Ding LW, Sun QY, Tan KT, Chien W, Mayakonda A, Yeoh AEJ, et al. Mutational Landscape of Pediatric Acute Lymphoblastic Leukemia. Cancer Res. 2017 Jan 15;77(2):390-400.
18. Roberts KG, Li Y, Payne-Turner D, Harvey RC, Yang YL, Pei D, et al. Targetable kinase-activating lesions in Ph-like acute lymphoblastic leukemia. N Engl J Med. 2014 Sep 11;371(11):1005-15.
19. Stam RW, den Boer ML, Schneider P, Meier M, Beverloo HB, Pieters R. D-HPLC analysis of the entire FLT3 gene in MLL rearranged and hyperdiploid acute lymphoblastic leukemia. Haematologica. 2007 Nov;92(11):1565-8.
20. Malinowska-Ozdowy K, Frech C, Schönegger A, Eckert C, Cazzaniga G, Stanulla M, et al. KRAS and CREBBP mutations: a relapse-linked malicious liaison in childhood high hyperdiploid acute lymphoblastic leukemia. Leukemia. 2015 Aug;29(8):1656-67.
21. Zhang J, Mullighan CG, Harvey RC, Wu G, Chen X, Edmonson M, et al. Key pathways are frequently mutated in high-risk childhood acute lymphoblastic leukemia: a report from the Children's Oncology Group. Blood. 2011 Sep 15;118(11):3080-7.
22. Liu Y, Easton J, Shao Y, Maciaszek J, Wang Z, Wilkinson MR, et al. The genomic landscape of pediatric and young adult T-lineage acute lymphoblastic leukemia. Nat Genet. 2017 Aug;49(8):1211-8.
23. Zhang Y, Zhang Y, Wang F, Wang M, Liu H, Chen X, et al. The mutational spectrum of FLT3 gene in acute lymphoblastic leukemia is different from acute myeloid leukemia. Cancer Gene Ther. 2020 Feb;27(1-2):81-8.
24. Holmfeldt L, Wei L, Diaz-Flores E, Walsh M, Zhang J, Ding L, et al. The genomic landscape of hypodiploid acute lymphoblastic leukemia. Nat Genet. 2013 Mar;45(3):242-52.
25. Zhang H, Wang H, Qian X, Gao S, Xia J, Liu J, et al. Genetic mutational analysis of pediatric acute lymphoblastic leukemia from a single center in China using exon sequencing. BMC Cancer. 2020 Mar 12;20(1):211.
26. Zhang J, McCastlain K, Yoshihara H, Xu B, Chang Y, Churchman ML, et al. Deregulation of DUX4 and ERG in acute lymphoblastic leukemia. Nat Genet. 2016 Dec;48(12):1481-9.
27. de Smith AJ, Ojha J, Francis SS, Sanders E, Endicott AA, Hansen HM, et al. Clonal and microclonal mutational heterogeneity in high hyperdiploid acute lymphoblastic leukemia. Oncotarget. 2016 Nov 8;7(45):72733-45.
28. Lindqvist CM, Lundmark A, Nordlund J, Freyhult E, Ekman D, Carlsson Almlof J, et al. Deep targeted sequencing in pediatric acute lymphoblastic leukemia unveils distinct mutational patterns between genetic subtypes and novel relapse-associated genes. Oncotarget. 2016 Sep 27;7(39):64071-88.
29. Reshmi SC, Harvey RC, Roberts KG, Stonerock E, Smith A, Jenkins H, et al. Targetable kinase gene fusions in high-risk B-ALL: a study from the Children's Oncology Group. Blood. 2017 Jun 22;129(25):3352-61.
30. Paulsson K, Lilljebjörn H, Biloglav A, Olsson L, Rissler M, Castor A, et al. The genomic landscape of high hyperdiploid childhood acute lymphoblastic leukemia. Nat Genet. 2015 Jun;47(6):672-6.
31. Chen C, Bartenhagen C, Gombert M, Okpanyi V, Binder V, Rottgers S, et al. Next-generation-sequencing of recurrent childhood high hyperdiploid acute lymphoblastic leukemia reveals mutations typically associated with high risk patients. Leuk Res. 2015 Sep;39(9):990-1001.
32. Andersson AK, Ma J, Wang J, Chen X, Gedman AL, Dang J, et al. The landscape of somatic mutations in infant MLL-rearranged acute lymphoblastic leukemias. Nat Genet. 2015 Apr;47(4):330-7.

33. Safavi S, Olsson L, Biloglav A, Veerla S, Blendberg M, Tayebwa J, et al. Genetic and epigenetic characterization of hypodiploid acute lymphoblastic leukemia. Oncotarget. 2015 Dec 15;6(40):42793-802.
34. Spinella JF, Cassart P, Garnier N, Rousseau P, Drullion C, Richer C, et al. A novel somatic mutation in ACD induces telomere lengthening and apoptosis resistance in leukemia cells. BMC Cancer. 2015 Sep 7;15:621.
35. Zhang J, Ding L, Holmfeldt L, Wu G, Heatley SL, Payne-Turner D, et al. The genetic basis of early T-cell precursor acute lymphoblastic leukaemia. Nature. 2012 Jan 11;481(7380):157-63.
36. Frohling S, Scholl C, Levine RL, Loriaux M, Boggon TJ, Bernard OA, et al. Identification of driver and passenger mutations of FLT3 by high-throughput DNA sequence analysis and functional assessment of candidate alleles. Cancer Cell. 2007 Dec;12(6):501-13.
37. Vicente C, Schwab C, Broux M, Geerdens E, Degryse S, Demeyer S, et al. Targeted sequencing identifies associations between IL7R-JAK mutations and epigenetic modulators in T-cell acute lymphoblastic leukemia. Haematologica. 2015 Oct;100(10):1301-10.
38. Sandhofer N, Bauer J, Reiter K, Dufour A, Rothenberg M, Konstandin NP, et al. The new and recurrent FLT3 juxtamembrane deletion mutation shows a dominant negative effect on the wild-type FLT3 receptor. Sci Rep. 2016 Jun 27;6:28032.
39. Reindl C, Bagrintseva K, Vempati S, Schnittger S, Ellwart JW, Wenig K, et al. Point mutations in the juxtamembrane domain of FLT3 define a new class of activating mutations in AML. Blood. 2006 May 1;107(9):3700-7.
40. Schittenhelm MM, Yee KW, Tyner JW, McGreevey L, Haley AD, Town A, et al. FLT3 K663Q is a novel AML-associated oncogenic kinase: Determination of biochemical properties and sensitivity to Sunitinib (SU11248). Leukemia. 2006 Nov;20(11):2008-14.
41. Opatz S, Polzer H, Herold T, Konstandin NP, Ksienzyk B, Zellmeier E, et al. Exome sequencing identifies recurring FLT3 N676K mutations in core-binding factor leukemia. Blood. 2013 Sep 5;122(10):1761-9.
42. Yamamoto Y, Kiyoi H, Nakano Y, Suzuki R, Kodera Y, Miyawaki S, et al. Activating mutation of D835 within the activation loop of FLT3 in human hematologic malignancies. Blood. 2001 Apr 15;97(8):2434-9.
43. Clark JJ, Cools J, Curley DP, Yu JC, Lokker NA, Giese NA, et al. Variable sensitivity of FLT3 activation loop mutations to the small molecule tyrosine kinase inhibitor MLN518. Blood. 2004 Nov 1;104(9):2867-72.
44. Grundler R, Thiede C, Miething C, Steudel C, Peschel C, Duyster J. Sensitivity toward tyrosine kinase inhibitors varies between different activating mutations of the FLT3 receptor. Blood. 2003 Jul 15;102(2):646-51.
45. Armstrong SA, Mabon ME, Silverman LB, Li A, Gribben JG, Fox EA, et al. FLT3 mutations in childhood acute lymphoblastic leukemia. Blood. 2004 May 1;103(9):3544-6.
46. Janke H, Pastore F, Schumacher D, Herold T, Hopfner KP, Schneider S, et al. Activating FLT3 mutants show distinct gain-of-function phenotypes in vitro and a characteristic signaling pathway profile associated with prognosis in acute myeloid leukemia. PLoS One. 2014;9(3):e89560.
47. Spiekermann K, Bagrintseva K, Schoch C, Haferlach T, Hiddemann W, Schnittger S. A new and recurrent activating length mutation in exon 20 of the FLT3 gene in acute myeloid leukemia. Blood. 2002 Nov 1;100(9):3423-5.
48. Jiang J, Paez JG, Lee JC, Bo R, Stone RM, DeAngelo DJ, et al. Identifying and characterizing a novel activating mutation of the FLT3 tyrosine kinase in AML. Blood. 2004 Sep 15;104(6):1855-8.
49. Bagrintseva K, Schwab R, Kohl TM, Schnittger S, Eichenlaub S, Ellwart JW, et al. Mutations in the tyrosine kinase domain of FLT3 define a new molecular mechanism of acquired drug resistance to PTK inhibitors in FLT3-ITD-transformed hematopoietic cells. Blood. 2004 Mar 15;103(6):2266-75.
50. Kindler T, Breitenbuecher F, Kasper S, Estey E, Giles F, Feldman E, et al. Identification of a novel activating mutation (Y842C) within the activation loop of FLT3 in patients with acute myeloid leukemia (AML). Blood. 2005 Jan 1;105(1):335-40.

*__Tarlock, K__, Hansen ME, Hylkema T, Ries, R, Farrar JE, Guidry Auvil J, Gerhard DS, Smith MA, Davidsen TM, Gesuwan P, Hermida LC, Marra MA, Mungall A, Mungall K, Ma Y, Zong S, Long W, Boggon T, Alonzo TA, Kolb EA, Gamis AS, Meshinchi S. Discovery and Functional Validation of Novel Pediatric Specific FLT3 Activating Mutations in Acute Myeloid Leukemia: Results from the COG/NCI Target Initiative *https://ash.confex.com/ash/2015/webprogramscheduler/Paper80715.html*
